# Triage through telemedicine in paediatric emergency care—Results of a concordance study

**DOI:** 10.1371/journal.pone.0269058

**Published:** 2022-05-26

**Authors:** Angelika Beyer, Kilson Moon, Peter Penndorf, Thomas Hirsch, Uta Zahn-Tesch, Wolfgang Hoffmann, Holger N. Lode, Neeltje van den Berg

**Affiliations:** 1 Institute for Community Medicine, Section Epidemiology of Health Care and Community Health, University Medicine Greifswald, Greifswald, Germany; 2 Department for Paediatrics, Sana-Hospital Ruegen, Bergen, Germany; 3 Department for Paediatrics, University Medicine Greifswald, Greifswald, Germany; Kaohsuing Medical University Hospital, TAIWAN

## Abstract

**Background:**

In the German health care system, parents with an acutely ill child can visit an emergency room (ER) 24 hours a day, seven days a week. At the ER, the patient receives a medical consultation. Many parents use these facilities as they do not know how urgently their child requires medical attention. In recent years, paediatric departments in smaller hospitals have been closed, particularly in rural regions. As a result of this, the distances that patients must travel to paediatric care facilities in these regions are increasing, causing more children to visit an ER for adults. However, paediatric expertise is often required in order to assess how quickly the patient requires treatment and select an adequate treatment. This decision is made by a doctor in German ERs. We have examined whether remote paediatricians can perform a standardised urgency assessment (triage) using a video conferencing system.

**Methods:**

Only acutely ill patients who were brought to a paediatric emergency room (paedER) by their parents or carers, without prior medical consultation, have been included in this study. First, an on-site paediatrician assessed the urgency of each case using a standardised triage. In order to do this, the Paediatric Canadian Triage and Acuity Scale (PaedCTAS) was translated into German and adapted for use in a standardised IT-based data collection tool. After the initial on-site triage, a telemedicine paediatrician, based in a different hospital, repeated the triage using a video conferencing system. Both paediatricians used the same triage procedure. The primary outcome was the degree of concordance and interobserver agreement, measured using Cohen’s kappa, between the two paediatricians. We have also included patient and assessor demographics.

**Results:**

A total of 266 patients were included in the study. Of these, 227 cases were eligible for the concordance analysis. In n = 154 cases (68%), there was concordance between the on-site paediatrician’s and telemedicine paediatrician’s urgency assessments. In n = 50 cases (22%), the telemedicine paediatrician rated the urgency of the patient’s condition higher (overtriage); in 23 cases (10%), the assessment indicated a lower urgency (undertriage). Nineteen medical doctors were included in the study, mostly trained paediatric specialists. Some of them acted as an on-site doctor and telemedicine doctor. Cohen’s weighted kappa was 0.64 (95% CI: 0.49–0.79), indicating a substantial agreement between the specialists.

**Conclusions:**

Telemedical triage can assist in providing acute paediatric care in regions with a low density of paediatric care facilities. The next steps are further developing the triage tool and implementing telemedicine urgency assessment in a larger network of hospitals in order to improve the integration of telemedicine into hospitals’ organisational processes. The processes should include intensive training for the doctors involved in telemedical triage.

**Trial registration:**

DRKS00013207.

## Introduction

In Germany, parents or caregivers can take children or adolescents with acute or subacute emergencies to the closest emergency room (ER). ERs are housed within hospitals. If the hospital has a paediatric department, then it will also have a paediatric ER (paedER). If it does not, the ER will cater to patients of all ages. However, it will not operate with specialised paediatric staff.

Some paediatric departments in smaller hospitals have been forced to close due to economic challenges and a lack of specialist staff. This is more frequently to occur in regions with a low population density. As a result of the closure of these departments, the geographic proximity to hospitals with a paedER is decreasing in many sparsely-populated areas in Germany. As a consequence, children and adolescents are more frequently treated at adult ERs. In some cases, patients with a low level of urgency, particularly older children, can receive adequate treatment on these wards. However, in other cases, a paediatric specialist is required to ensure appropriate diagnosis and treatment.

Telemedicine is a promising option if there is no paediatric specialist on-site. However, little use has been made of telemedicine and its systematic evaluation in acute paediatric care in Germany.

### General and paediatric telemedicine

An early study conducted in Canada in 1980 showed that telephone screenings could reduce the number of visits to paediatric emergency departments [1]. Since 2005, Canada has implemented various validated video-based training programs in telemedical triage for medical doctors and advanced nurses. These programs were implemented in order to reduce the number of visits to emergency departments, as well as unnecessary transfers to remote specialist clinical departments for both adult and children’s medicine [2–5]. The outcome was largely positive in regard to using telemedicine to reproduce primary diagnoses of acute childhood illness in cases where patients had acute problems with their upper respiratory tract or ears. There was a subsequent push to develop remotely-managed medical instruments (e.g. video-capable otoscopes) [6–9].

Multiple countries have established telemedicine centres to support paediatric acute healthcare, including: Australia [10, 11], USA [12–14], Canada [15–17] and Israel [18]. These centres assist with the adequate allocation of patients, especially in rural or remote regions.

The use of telemedicine technology remains limited and difficulties have been reported, due to frequent problems with technology and implementation [19]. Physicians also face various challenges when using telemedicine to make decisions about treatment [20]. Physicians cannot use a number of diagnostic techniques due to the differences between telemedicine and on-site assessment. They cannot use specific medical equipment or techniques that require them to use their sense of smell or touch. Hence, physicians must be trained in order to obtain the special expertise, qualities and skills required to make telemedicine diagnoses. In 2021, Weigel et al. noted that telemedical design choices also have implications for evaluative measures. It is important that designs reflect the model’s purpose and focus on the intended recipient of the tele-service [21].

### Triage-related telemedicine

A retrospective analysis examined 399 paediatric cases that involved telemedical triage. During this analysis, the appropriateness of the diagnosis and the treatment decisions made, based on the documented triage procedures and written documentation from other doctors, were assessed. The diagnosis was judged as appropriate in 98.5% and the decisions in 92% of the cases. The study concluded that telemedical triage results in a high level of patient safety [22]. However, there are some challenges in triage-related telemedicine, for example, adequate pain assessment in a telemedicine context, especially in vulnerable patients who cannot express themselves [23–29]. Other limitations include the bounded feasibility of urgency assessments and the assessment of the need for on-site treatment for brain injuries or concussions (e.g. for specialised neurological care or intervention) [15, 16, 30].

To summarise, there is limited data available in regard to the use of telemedicine solutions in acute paediatric healthcare, especially in Germany. More research is needed in order to advance the use of telemedicine in triage paediatric healthcare. In this study, we examined whether telemedicine can be used to assess the urgency of cases in acute paediatric healthcare. We investigated the extent to which the results of triage assessments carried out via video conferencing, based on a German adaptation of the Paediatric Canadian Triage and Acuity Scale (PaedCTAS) [31], correspond with the results of on-site triage in a paedER.

## Materials and methods

### Design and setting

A multicentre concordance study including with five paedERs, all in rural regions, was carried out. The participating paedERs were widely dispersed within the German Federal State of Mecklenburg-Western Pomerania. Children and adolescents under the age of 18, who visited one of the participating paedERs, received a standardised triage procedure. The on-site doctor conducted the first triage procedure. This was followed by a telemedicine doctor in another hospital, via video conference. The telemedical triage was not relevant for the treatment; the on-site doctor had the authority in regard to treatment and retained sole responsibility for the patient. The results of both triage procedures for each individual patient were analysed. The degree of concordance and the interobserver agreement, measured using Cohen’s kappa, were the primary outcome. Patient and assessor demographics have been reported.

### The triage tool

In the absence of an existing video-based and standardised triage instrument, a translated and adapted version of the Paediatric Canadian Triage and Acuity Scale (PaedCTAS) [31] was used only in this study. It was implemented within a newly developed data collection tool. This data collection tool was not integrated into the participating hospitals’ patient information or documentation systems. It was implemented and evaluated independently in the participating paedERs.

### Data collection

The following steps were taken in the study: first, the on-site doctor or the supporting person created a case report in the data collection tool. The case report documented the patient’s socio-demographic data, a brief medical history and the result for each triage parameter. The telemedicine doctor used the same case report form in their documentation but could only see socio-demographic data. The data collection tool was available via a secure virtual private network (VPN).

The first of the triage parameters within our data collection tool was concerned with the initial impression of the patient’s condition, in regard to the child’s general appearance, breathing and circulation. This is called the Paediatric Assessment Triangle, known as the “critical look” in the PaedCTAS’ improved guidelines [31]. If any of these criteria deteriorated in the paedER, the standardised triage procedure was discarded in favour of promptly initiating diagnosis and treatment. Otherwise, each of the twenty parameters in the PaedCTAS was used. The parameters generated an urgency rating from level I to IV: I = acute vital threat; II = emergency; III = urgent; IV = less urgent (unlike the PaedCTAS, the German instrument did not differentiate between level IV = less urgent and V = not urgent due to similar clinical consequences). After reviewing these twenty parameters, the individual parameter with the highest urgency level determined the overall urgency level.

Some of the triage parameters could not be determined by video conference and were measured on-site by a nurse or a medical student (respiratory rate, heart rate, oxygen saturation and body temperature). These four parameters were reported verbally to the telemedicine doctor by the supporting on-site staff. The telemedicine doctor could also ask for the measurement to be repeated. Other parameters were assessed independently by the telemedicine doctor (e.g. pain, the Glasgow Coma Scale parameters, traumatic injuries). The twenty parameters reviewed and type of survey used are listed in Table 1.

**Table 1:** Parameters of the triage tool adapted from Warren et al. 2008 [31].

No.	Parameter^a^	Assessment (type of survey)^b^
1	Initial assessment (filter question)	on-site
2	Respiratory rate (breaths per minute)	on-site
3	Oxygen saturation	on-site
4	Heart rate (pulse rate per minute)	on-site
5	Open eyes	on-site and telemedicine doctor
6	Verbal communication	on-site and telemedicine doctor
7	Motor response	on-site and telemedicine doctor
8	Glasgow Coma Scale	score from items 5–7
9	Body temperature (centigrade)	on-site
10	Temperature classification	on-site and telemedicine doctor
11	Pain	on-site and telemedicine doctor
12	Trauma injuries	on-site and telemedicine doctor
13	Concern for patient’s welfare	on-site and telemedicine doctor
14	Paediatric disruptive behaviour (behavioural disorder)	on-site and telemedicine doctor
15	Floppy child	on-site and telemedicine doctor
16	Paediatric gait disorder or painful walk	on-site and telemedicine doctor
17	Stridor	on-site and telemedicine doctor
18	Apneic spells	on-site and telemedicine doctor
19	Inconsolable crying	on-site and telemedicine doctor
20	Congenital problem	on-site and telemedicine doctor

^**a**^ The overall urgency level corresponds to the highest urgency level assigned when assessing the individual items.

^**b**^ Each on-site measurement could be repeated at the request of the telemedicine doctor.

We used the Visual Analog Scale (0 = no pain to 10 = strongest imaginable pain) to assess pain. This scale is usually used in PaedCTAS [31] and as per local routine. As this instrument is not suitable for younger children, pain was assessed using the KUS-Scale (*Kindliche Unbehagen- und Schmerzskala nach Büttner* [Children and Infants Postoperative Pain Scale (CHIPPS) according to Buttner]) [32] for children under 4 years of age. For children between the ages of 4 and 7, the MOPS (Modified Objective Pain Scale) [33] was used. A German translation of the Neonatal Infant Pain Scale, observing their facial expression, crying/screaming, breathing, arm/leg movement and alertness [34] was used for newborns.

### Telemedicine

The same video conferencing system (Cisco Webex^TM^) was set up in each of the participating hospitals for the internet-based connection. The system had to meet the following requirements:

- high video and audio quality
- a high-resolution mobile camera with high optical zoom function on the patient’s side
- the on-site nurse and the remote telemedicine doctor needed to be able to control and manoeuvre the camera

### Selection of participants and workflow

#### Study site

The hospitals that participated in the study were located in the German Federal State of Mecklenburg-Western Pomerania and had a working paedER. In total, five hospitals took part in the study, four of them were responsible for recruiting patients. All five hospitals carried out triage for the entire duration of the study. Nineteen medical doctors participated in the study, mostly trained paediatric specialists. Some acted as both an on-site and telemedicine doctor. Fourteen different doctors were involved in the on-site triage. Of these, ten were paediatricians that assessed 75.8% of the cases in the study (n = 172, min = 1, max = 123). The other four doctors were paediatricians undertaking their training, who assessed the remaining 24.2% of cases. Eighteen doctors provided the telemedical triage. Of these, fourteen were paediatricians, who assessed 87.7% of the cases (n = 199, min = 1, max = 65). The other four doctors were paediatricians undertaking their training and assessed the remaining 12.3% of cases. Some doctors acted as both an on-site and telemedicine doctor for separate patients.

#### Patients who participated

Inclusion criteria: paediatric patients under the age of 18, who had visited the paedER in one of the participating hospitals under their parents’ or caregivers’ own initiative (without asking a health professional or calling a nationwide central telephone number for advice beforehand) were eligible to participate in the study. Exclusion criteria: in order to counteract concerns that the telemedicine project could lead to a deterioration in the quality of treatment, patients were excluded from the study if they showed signs of a very critical health condition upon arrival at the paedER. These conditions included serious problems with patients’ general appearance, breathing and circulation and if they were at risk of acute further deterioration. Patients were recruited at times when a telemedicine doctor was available.

#### Workflow

The paediatric patients and their parents or caregivers were informed about the project and were asked to sign a written consent form. After consent was obtained, the on-site paediatrician performed the standardised triage procedure and recorded a short medical history. In Germany, only a medical doctor can determine the next steps in terms of treatment. After this, the on-site paediatrician left the room and the patient was connected to the telemedicine doctor located in one of the partner hospitals. The telemedicine doctor–also a paediatrician–then carried out the triage procedure via video conference, supported on-site by a nurse or an internship year (final year) medical student. The individuals who provided support received an introduction to the telemedicine requirements before the procedure but did not have any real-life experience with telemedicine functions and procedures. The on-site support was required to use the technical equipment and to measure and transmit several triage parameters (see section Triage Tool). After completing the telemedical triage, the on-site doctor carried out the treatment as usual. The on-site and telemedicine doctor did not have access to the other’s assessment of the patient.

### Data analysis

The data was analysed using Stata® Version 14.2 (Copyright 1985–2015 StataCorp LP, StataCorp 4905 Lakeway Drive College Station, Texas 77845 USA 2015). Descriptive statistics (frequency distributions) were used for the data pertaining to the urgency assessments in triage procedures. A 95%-CI is reported for the age of the patients. This also applies to the interobserver agreement, which was calculated to measure concordance between on-site and telemedicine assessments (squared weighting for four categories and two assessors). The degree of concordance was classified based on the ranges suggested by Landis & Koch: < 0 = poor, 0.01–0.20 = slight, 0.21–0.40 = fair, 0.41–0.60 = moderate, 0.61–0.80 = substantial, 0.81–1.00 = (almost) perfect [35]. The urgency level IV (less urgent) was assumed for measurements or assessments that were missing (or "does not apply" entries).

### Ethical consideration

In 2015, the University Medicine Greifswald’s Clinical Ethics Committee (protocol number BB125/14, decision letter dated: 2015/02/17) approved the study. We applied for two amendments due to organisational changes, which were also confirmed (BB125/14a, decision letter dated: 2016/09/06 and BB125/14b, decision letter dated: 2017/09/22).

## Results

**Fig 1:**
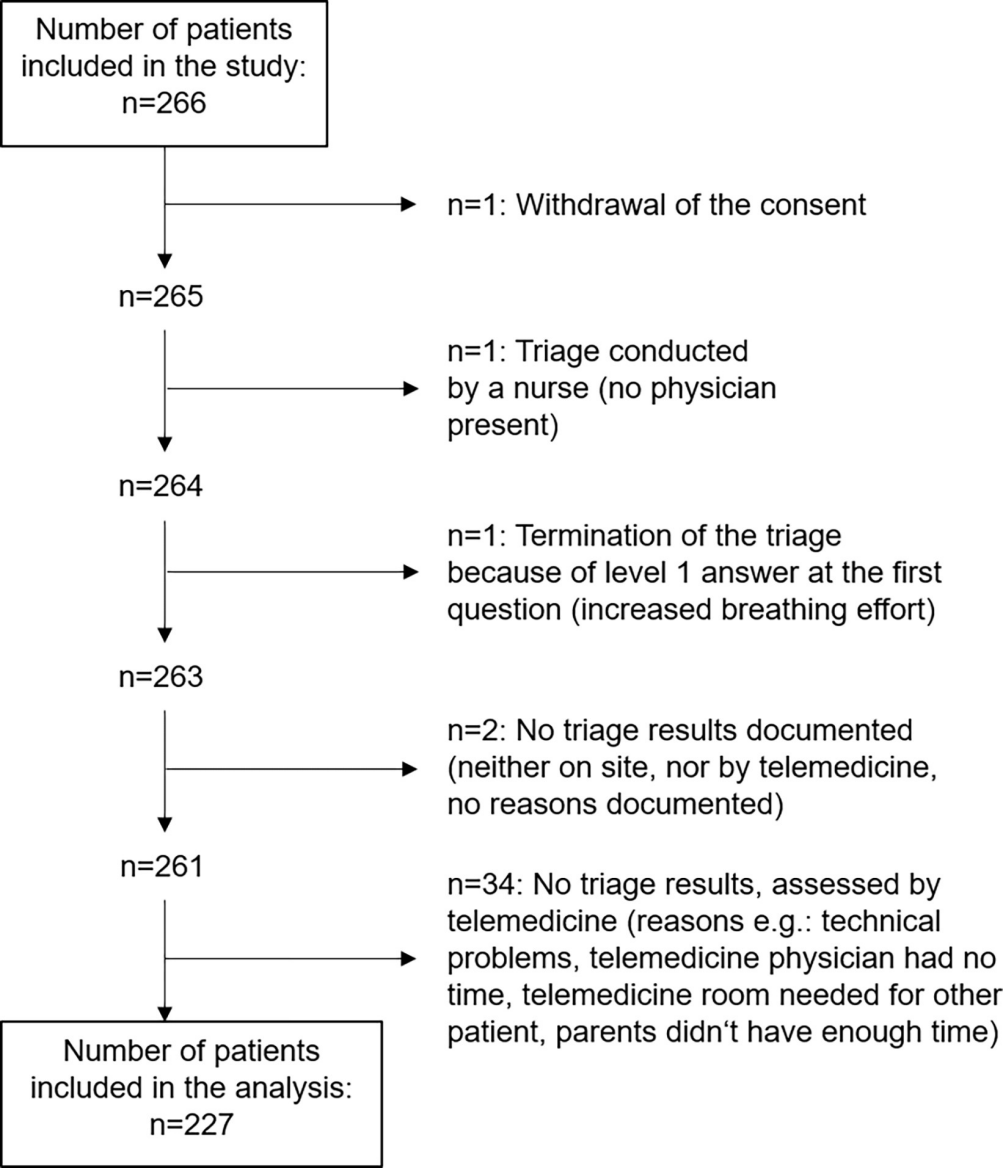
Flow diagram of the study.

A total of 266 patients were recruited in four hospitals between May 2015 and September 2019. Of these, the triage results of 85.3% of the patients (n = 227) were included in the analysis of the main outcome (see Fig 1).

The hospitals that took part in the study recruited between one and 124 patients each. The paediatric department in two of these hospitals was closed shortly after the start of the study. This meant that the recruitment ended after admitting just one and ten patients to the respective hospitals.

49.8% of the patients (n = 113) were male. The average age of the patients was 4.74 years (95% CI: 4.58–4.89). The largest subgroup of patients was between one and three years old (n = 81; 35.7%), followed by those over 7 years old (n = 78; 34.4%). 22 children (9.7%) were less than one year old.

**Fig 2:**
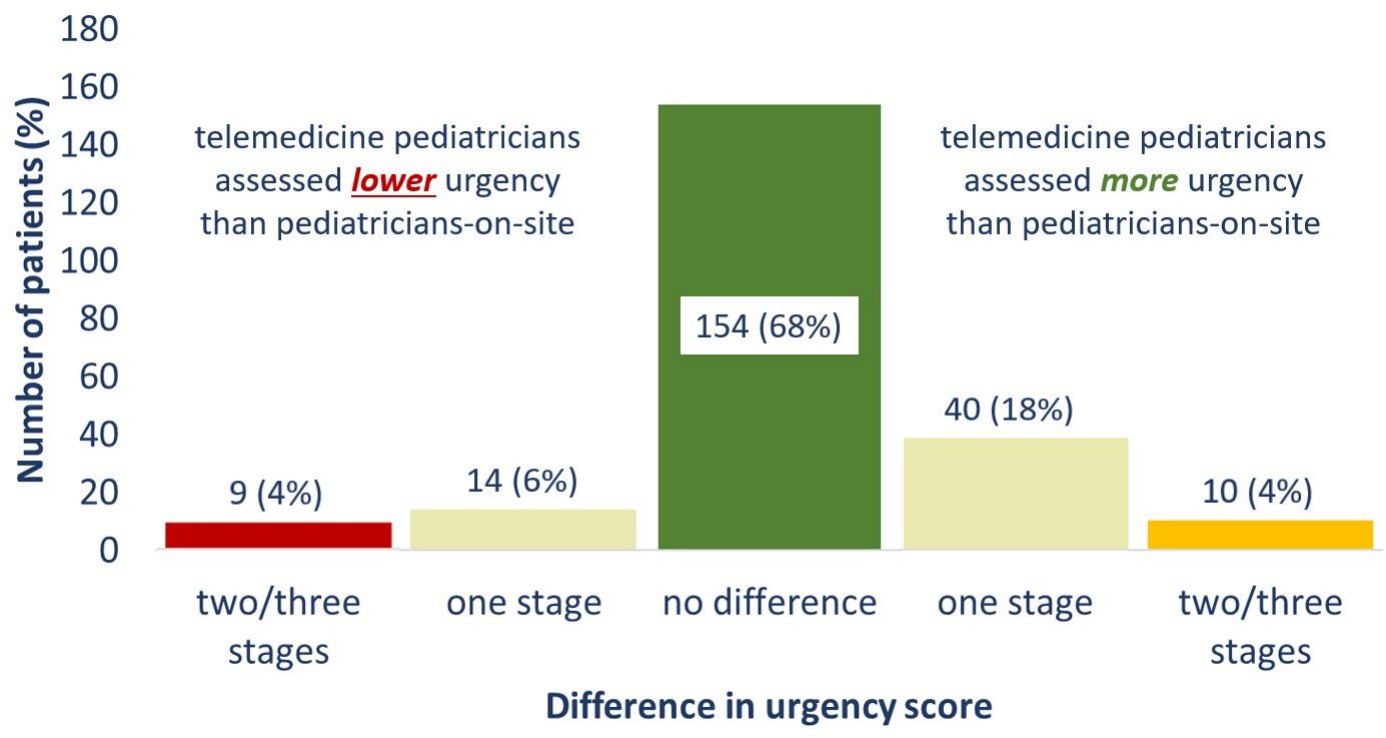
Overall urgency levels—differences between the on-site and telemedicine assessments.

In 154 cases (67.8%), the urgency level determined on-site corresponded exactly to the assessments made via telemedicine. In 23 cases (10.1%), the urgency level ascertained by the telemedicine doctor was lower than that of the on-site doctor (undertriage). Among those, there were 9 cases (4.0%) in which the difference was greater than one urgency level. In 50 cases (22.0%), the telemedicine doctor rated the urgency higher than the on-site doctor (overtriage), whereby there were 10 cases (4.4%) with a difference greater than one urgency level (see Fig 2).

Cohen’s squared weighted kappa was 0.64 (95% CI: 0.53–0.75). This value has been classified as a substantial agreement [35] and is highly significant (p<0.001).

The urgency levels assigned by the on-site doctor were: in 81 cases (35.7%), urgency level IV; in 82 cases (36.1%), urgency level III; in 43 cases (18.9%), level II and in 21 cases (9.2%), level I without risk of further acute deterioration (see Table 2).

**Table 2:** Contingency table for the overall urgency levels.

		Urgency level assessed by telemedicine doctor				
		I	II	III	IV	Total
Urgency level assessed by on-site doctor	I	**16**	0	3	2	21
	II	4	**30**	5	4	43
	III	2	15	**56**	9	82
	IV	2	6	21	**52**	81
	Total	24	51	85	67	227

I = acute vital threat; II = emergency; III = urgent; IV = less urgent

Cohen’s squared weighted kappa: 0.64 (95% CI: 0.53–0.75, significance = <0.001).

Table 3 shows the results of the analysis of the individual items which make up the triage tool. Differences were found for the items that were only assessed on-site, which were only repeated at the request of the telemedicine doctor. These differences were noted in oxygen saturation (deviation in 3 cases), in respiratory rate (deviation in 22 cases, 12 of which had a higher urgency assessment provided by the telemedicine doctor), in heart rate (deviation in 27 cases, 13 of which had a higher urgency assessment provided by the telemedicine doctor). The results also differed in other items that could be assessed independently by the telemedicine doctor. In 49 cases (22%), there were differences between on-site and telemedicine observations when assessing pain intensity. In 29 cases thereof (13%), the telemedicine assessment produced a higher urgency than the on-site assessment. In 20 cases (9%), the reverse was true. In terms of the acute trauma parameter, there were different urgency levels determined in 10 cases (4%), of which 7 cases (3%) showed a higher urgency in the telemedical triage. The accordance differed for the Glasgow Coma Scale in 3 cases and was more than 99% for the other 8 items, as shown in Table 3.

**Table 3:** Comparison of urgency level assessments for the individual items in the triage tool (n = 227 cases).

Parameter	Number of cases without a difference between the observations (percent)	Number of cases with a difference between the observations (percent)	Telemedical triage less urgent than on-site assessment (undertriage)		Telemedical triage more urgent than on-site assessment (overtriage)	
			>1 level difference	1 level difference	>1 level difference	1 level difference
Parameters that had to be measured on-site, repeated only at the request of the telemedicine doctor						
Respiratory rate	205 (90.3)	22 (9.7)	9	1	6	6
Oxygen saturation	224 (98.7)	3 (1.3)	1	-	2	-
Heart rate	200 (88.1)	27 (11.9)	5	9	5	8
Temperature	194 (85.5)	33^a^ (14.5)	-	-	-	-
Parameters assessed on-site and independently by the telemedicine doctor						
Glasgow Coma Scale (total score)	224 (98.7)	3 (1.3)	2	-	1	-
Temperature classification	227 (100)	0	-	-	-	-
Pain	178 (78.4)	49 (21.6)	1	19	2	27
Trauma injuries	217 (95.6)	10 (4.4)	3	-	7	-
Concern for patient’s welfare	227 (100)	0	-	-	-	-
Paediatric disruptive behaviour (behavioural disorders)	227 (100)	0	-	-	-	-
Floppy child	227 (100)	0	-	-	-	-
Paediatric gait disorder or painful walk	226 (99.6)	1 (0.4)	-	1	-	-
Stridor	225 (99.1)	2 (0.9)	-	2	-	-
Apneic spells	225 (99.1)	2 (0.9)	-	2	-	-
Inconsolable crying	226 (99.6)	1 (0.4)	-	1	-	-
Congenital problem	227 (100)	0	-	-	-	-

^**a**^ Differences in the measured value but not in the level of urgency, as this was assessed in “Temperature classification”

## Discussion

The comparison of on-site versus telemedical triage showed concordance in the overall urgency level in two thirds of cases. Thus, in our sample there was substantial concordance. However, in one third of the cases there was a difference between the overall-items-urgency-level provided by the on-site and telemedicine doctors (see Fig 2). Three different points will be discussed in this paper: first, the direction of the deviation (known as overtriage and undertriage). The second point relates to the extent of this difference and what can be done to reduce both overtriage and undertriage in a follow-up study. The third point addresses time-related differences. In our study, differences in urgency level had *no* effect on the patients, as the treatment decision was the on-site doctor’s responsibility and the telemedicine assessment was an additional assessment.

First, the direction of the deviation is important for the way in which the results were interpreted. In our study, we defined cases that were assigned a higher urgency level by the telemedicine assessment than the on-site assessment as overtriage. We found overtriage in 50 of the 73 cases that had been assigned different levels, this affected every fifth case in our study. This is a high proportion; however, these cases are clinically safe. A higher level of urgency does not result in the patient being placed in a dangerous situation, due to problems that have not been recognised. In our study, overtriage had no effect, as the treatment decision was the on-site doctor’s responsibility and the telemedicine assessment was an additional assessment. Nevertheless, in a real healthcare setting, overtriage could lead to unnecessary transfers to another hospital or unnecessary examinations. However, other studies have shown that telemedicine reduced the number of transfers and costs while still ensuring patients’ safety [10–12, 14, 15, 18]. Cases in which the telemedicine doctor assigned a lower level of urgency than the on-site doctor (undertriage) should be evaluated more critically. In this study, this affected every tenth case (n = 23). Undertriage in this study had no effect on the patients. However, analysing the individual triage items has been extremely informative, as this has provided a more detailed picture of the individual triage and possible clinical consequences of any discrepancies. Furthermore, a detailed consideration of the triage parameters can reveal the triage tool’s strengths and limitations.

Second, the extent of the differences was considered. In 24% of the cases (n = 54, thereof overtriage: n = 40 and undertriage: n = 14), there was a difference of one level. We noted that most of these cases in our study involved respiratory or heart rate issues, pain and inconsolable crying. It should be noted that these parameters can change considerably over short periods of time. However, in five of the cases, the telemedicine doctor did not see problems that the on-site-doctor, who was responsible for the first assessment, had seen (gait disorder or painful walk, stridor or apneic spells). In 8% of cases (n = 19, thereof overtriage: n = 10 and undertriage: n = 9), there was a difference of two or three levels. The differences in the urgency assessment were also related to respiratory rate, heart rate or pain, oxygen saturation, the Glasgow coma scale and trauma injuries. This study confirms that trauma cases should be assessed very carefully when employing telemedicine [15, 16, 30]. Undertriage is a serious problem that requires specific attention in a follow-up study. Undertriage may be mitigated by increasing medical staff training and providing clearer category definitions in the triage tool. These strategies may contribute to telemedical triage being implemented in everyday clinical practice.

Third, some differences, especially those related to respiratory rate, heart rate or pain, may be caused by the workflow. This could be caused by time delays between the measurement performed by the on-site doctor or nurse and the telemedicine doctor. These time delays were not documented accurately, due to technical and organisational problems. It is likely that the differences stem from time-related change in objectively measured vital signs and are not the result of a disagreement between the two physicians. The on-site triage was always conducted first for organisational reasons. Ideally, both assessments would have been carried out simultaneously, but this was not an option during this project.

Additionally, we looked for patterns with respect to the qualifications of the doctors who participated in this study. Neither overtriage nor undertriage triage occurred more often when the urgency assessment was conducted by doctors who had not completed specialist paediatric training. We believe that the lack of experience with telemedical triage, which applied to nearly all of the doctors participating in this research, may have led to some of the unexplained differences.

The majority of the differences occurred in the assessment of pain severity. Powell et al. discussed the difficulty of determining whether pain is clinically significant [36]. It is well documented that parental assessments of their child’s pain differs from what is reported by the children themselves [37]. Young children’s pain can only be measured by external assessment. It should be noted that the original 11-step visual analogue scale in Germany is normally only used for the self-assessment of older children by someone who is trained to do so. Parents may have been asked to provide an assessment by the telemedicine doctor, however, they lack the experience required to make a reliable judgement using this instrument. Furthermore, the number of steps in the 11-step scale had to be reduced in order to align the scale with the three-step urgency level used in both the PaedCTAS and in our own triage tool. To the best of our knowledge, there is still no validation of this 3-stage pain measurement. The difficulty of reliably measuring pain can be found in many studies [27, 38, 39]. Children’s sensitivity to pain can change over short periods of time, for example if they are being distracted or entertained [25, 28, 38], which were not used in this study. Most of the differences in pain measurement amounted to one urgency-level (n = 30). We assume that most discrepancies are due to the two paediatricians’ subjective assessment, as well as the time delay, rather than the telemedicine situation itself. This assumption is based on the fact that the differences in triage relating to pain went in both directions; both undertriage and overtriage occurred. This assumption requires further studies but it confirms the claims in Weigel’s publication, which describes building standardised metrics in tele-emergency models as a special challenge [21].

This study has some limitations. We used a modified triage tool. The number of urgency levels was reduced compared to the PaedCTAS (from 5 in the original tool to 4 in our tool). The triage tool was also developed for triage in children and adolescents, but not for telemedical use. Telemedical triage lacks some of the dimensions of on-site examination (e.g. three-dimensional observation of the patient, smell, touch, small colour changes on the skin that may not be visible on a monitor and limited perception of the environment). Therefore, it is crucial that the triage parameters are clearly described and can be documented easily and accurately. As this project was implemented in a real healthcare setting, the processes could not be completely standardised, some of the organisational processes had to be adapted.

We assume an information bias caused by time delay in the results because of the fixed order of the triage assessments and the time gap between the two observations. As described earlier, in some cases there were longer intervals between the observations. This could mean that differences between the observations were a consequence of actual changes in the patient’s health status. Different triage categories may be caused by time-related changes in the health situation as the time between the assessments varied due to the fact that the study was implemented in a real hospital setting. Only 3 out of the 20 triage-parameters did not change over time (trauma-injuries, apneic spells and congenital problems). This observer bias can be addressed in future studies by conducting simultaneous patient assessments by the on-site and the telemedicine doctor.

An observer bias could also result from the different attitudes, qualifications and experience of the doctors involved [19, 20].

There is likely to be a selection bias, as the patients included in this study do not represent all patients in a paediatric emergency room. Patients who were admitted by ambulance were not included in the study due to the severity of their illness. The majority of the patients had no serious illness or very urgent need for treatment. In the design of the study, there were concerns that the telemedicine project could lead to a deterioration in the quality of treatment, as the start of the treatment could be delayed. Thus, there is a clear selection bias, as only patients who were not brought in by ambulance were included. Another selection bias arises from the fact that a telemedicine doctor was not always available. It is possible that a selection bias was caused by undocumented data, so that these cases could not be compared. In this respect, there were major organisational problems.

As we did not have a priori assumption about the possible degree of concordance between the observations, we did not calculate the number of cases. Instead, we recruited as many consecutive patients as possible within the project period.

A consequence of the limitations is that the results cannot be generalised for all patients and situations in a paediatric emergency room.

On the other hand, the study has clear strengths. The study presents the typical patient group that comes to a paedER, as well as increasingly to hospitals that do not have a paediatric department. A challenge, and a strength of the study setting, was the need to implement telemedical triage within the framework of the hospitals’ existing organisational processes. This enabled us to observe organisational and technical problems and barriers during the implementation. The setting around routine care was a real challenge, e.g. oftentimes the telemedicine paediatrician was the only doctor on the floor and too busy to perform telemedical triage in addition to their other duties. This also occurred with on-site nursing staff. This problem could be solved by explicitly assigning telemedicine tasks in the roster. Each problem provided us with valuable information about implementing telemedicine concepts and transferring it to other settings and regions.

There are aspects we can improve in a following study, for example, more intensive training for the doctors involved, choosing a different or modified triage tool for telemedicine use, and, if possible, avoiding the time delays between repeated assessments by means of simultaneous on-site and telemedicine assessment.

## Conclusion

Our study showed that telemedical triage in paediatric acute care is a promising method in terms of supporting the healthcare system for acutely ill paediatric patients in ERs that do not have on-site paediatric expertise. No child was at risk as a result of the study as the telemedical triage was an additional assessment to the on-site assessment they had already received. With the increasing number of closures of paedERs in rural Germany, telemedicine methods may support paediatric acute care in these regions. Further studies that use optimised tools and processes, as well as multicentre and multicountry studies, studies in settings that already use telemedicine or a comparison of different triage systems, are needed to improve the quality and integration of telemedicine into the existing workflows in hospitals and ERs.
